# Supplementary material for: Identification of photoperiod-induced specific miRNAs in the adrenal glands of Sunite sheep (Ovis aries)
Source: Front Vet Sci. 2022 Jul 22;9:888207. doi: 10.3389/fvets.2022.888207 (PMC9354845; doi:10.3389/fvets.2022.888207)
Supplement: Supplementary file 1 [file Data_Sheet_1.ZIP › Supplementary file/miRDeep2 file.zip!/miRDeep2 file/Novel_Structure/LP42A2/Novel_441.pdf]

Provisional ID	:	NC_019467.2_18592
Score total	:	1.4
Score for star read(s)	:	-1.3
Score for read counts	:	-0.8
Score for mfe	:	2
Score for randfold	:	1.6
Score for cons. seed	:	
Total read count	:	10
Mature read count	:	7
Loop read count	:	3
Star read count	:	0

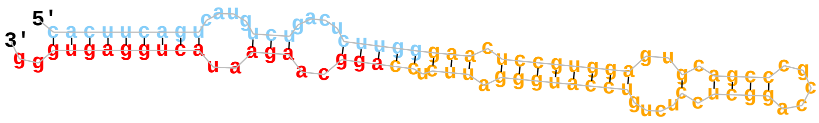

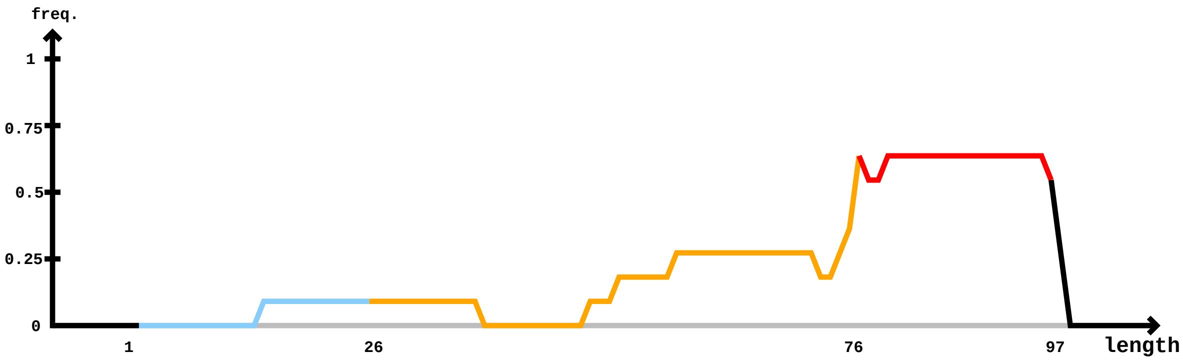

Star Mature

```
5'- gccaagucacuucagucaugucugacucuugggaacuccguggagugcagcccgccaggcuccucuguccaugggauucuccaggcaagaauacuggagugggguugccauuuc   -3'    exp
    (.(((.((((((((((....(((....((((((((.((((((((..(.((((.....)))).)....)))))))).))).)))))..)))..))))))))))).))).).....   reads       mm         sample
    ....................ucugacucuugggaacuccgugg......................................................................   1           0          seq
    ......................................................ccaggcuccucuguccaugggauucucUa..............................   1           1          seq
    .........................................................ggcuAcucuguccaugggauu...................................   1           1          seq
    ...............................................................ucuguccaCgggauucuccagg............................   1           1          seq
    ................................................................................ccaggcaagaauacugAagugggu.........   1           1          seq
    .................................................................................caggcaagaauacuggaguggg..........   1           0          seq
    ..................................................................................aggcaagaauacuggaguggg..........   3           0          seq
    .....................................................................................caCgaauacuggagugg...........   1           1          seq
    .....................................................................................caagaauacuggagugggu.........   1           0          seq
```
